# Other Universities

**Published:** 1907-09-07

**Authors:** 


### OTHER UNIVERSITIES.

### The University of Birmingham (Faculty of Medicine).

In order to obtain the degrees of M.B. and Ch.B. five examinations have to be passed. The first is in chemistry, physics, and elementary biology; the second in anatomy and physiology; the third in pathology, bacteriology, and materia medica and pharmacy; the fourth in forensic medicine, toxicology, and public health; and the fifth, or final, in medicine, surgery, midwifery, gynæcology, therapeutics, mental diseases, and ophthalmology. The University also grants degrees in dental surgery, B.D.S. and M.D.S., and a diploma (L.D.S.), and a diploma (D.P.H.) and a degree (B.Sc.) in public health. The General Hospital and the Queen's Hospital, containing between them upwards of 400 beds, are amalgamated for the purpose of clinical instruction, which is carried on under the direction of the Birmingham Clinical Board.

### University of Durham.

For students who intend to graduate at the Durham University it is necessary that one of the five years of professional education should be spent in attendance at the College of Medicine, Newcastle-upon-Tyne. During the year so spent the student must attend the medical and surgical practice and clinical lectures at the Infirmary, which contains over 400 beds. No residence at Durham is necessary. The remaining four years of the curriculum may be spent at Newcastle-upon Tyne or at one or more of the recognised medical schools.

### University of Edinburgh.

To obtain the Edinburgh medical degree it is necessary to pass the Preliminary Examination or its recognised equivalent, and to pass four professional examinations. The first is in botany, zoology, physics, and chemistry; the second in anatomy, and physiology, the third in pathology, materia medica, and therapeutics, and the final in surgery, clinical surgery, medicine, clinical medicine, midwifery, clinical gynæcology, forensic medicine, and public health.

The degree of M.B. is not conferred separately from that of Ch.B. In order to obtain the degree it is necessary to spend at least two of the five years of medical study in the University of Edinburgh.

### University of Aberdeen.

The regulations are practically the same as those given for Edinburgh. Clinical instruction is obtained in the Royal Infirmary, which contains 250 beds, in the Sick Children's Hospital, and several other institutions, including the Royal Lunatic Asylum and the City Fever Hospital.

### The University of Sheffield.

*Degrees of Bachelor of Medicine and Bachelor of Surgery.*—A candidate for the degrees of M.B., Ch.B., shall produce certificates that he will have attained the age of 21 years on the day of graduation; that he has pursued the courses of study required by the University Regulations during a period of not less than five years subsequently to the date of his registration as a medical student by the General Medical Council, three of such years at least having been passed in the University, and one year at least having been passed in the University subsequently to the date of passing the First Examination. Three examinations must be passed.

The subjects of the First Examination are Chemistry, Physics, and Biology.

The subjects of the Second Examination are Anatomy, Physiology, Materia Medica, and Pharmacy.

The subjects of the Third and Final Examination are divided into two parts, namely, A (Forensic Medicine and Toxicology, Public Health, and Pathology and Morbid Anatomy), and B (Medicine, including Pharmacology and Therapeutics, Mental Diseases, and Diseases of Children, Surgery, Obstetrics, and Gynæcology). Candidates may present themselves for examination in both parts on the same occasion or separately, but Part A may not be passed before the completion of the fourth year of study. Candidates for the whole examination, or for Part B, must have completed the fifth year of study. Candidates for the degree of Doctor of Medicine must have passed the examination for the degrees of M.B., Ch.B., at least one year previously, must present a thesis embodying observations in some subject approved by the Professor of Medicine, and must pass an examination in the Principles and Practice of Medicine. Candidates for the degree of Ch.M. must have passed the examination for the degrees of M.B., Ch.B., at least one year previously, and must, since taking the degrees of M.B., Ch.B., have held for not less than six months a surgical appointment in a public hospital or other public institution affording full opportunity for the study of Practical Surgery. The subjects of examination are Systematic, Clinical, and Operative Surgery, Surgical Anatomy, Surgical Pathology, and Bacteriology.

